# Nutritional Strategies to Prevent Weight Gain and Obesity

**DOI:** 10.3390/nu15194180

**Published:** 2023-09-27

**Authors:** Margaret Allman-Farinelli

**Affiliations:** Sydney Nursing School, Faculty of Medicine and Health and Charles Perkins Centre D17, University of Sydney, Camperdown, NSW 2006, Australia; margaret.allman-farinelli@sydney.edu.au

It has now been 25 years since the World Health Organisation (Geneva, Switzerland) drew attention to the obesity epidemic (later becoming a pandemic) [1] and, ever since, researchers have been working to prevent populations from gaining excessive weight and being on the upward trajectory to obesity. Obesity is often labelled a ‘wicked’ problem or a grand challenge almost too complex to solve. However, there have been successes in programs for individuals and communities and policies that can benefit whole populations, and these should be celebrated. This does not mean we can be complacent, and new programs and policies must be researched for targeted populations in low- and middle-income countries, as well as high-income countries, to meet people’s needs in this decade after the COVID-19 disruption and beyond.

Programs should be designed for people targeting each stage of the lifecycle for the prevention of unhealthy weight gain. It is well recognised that the first 1000 days of a child’s life will determine their future development, health, and achievements [2]. Hence, it is important that we begin with the health of the mother and father. The high global prevalence of overweight and obesity has impacted maternal and infant health. Excessive weight gain during pregnancy above the suggested amounts for the normal weight (11 to 16 kg); overweight (7 to 11 kg); and obese (5 to 9 kg) categories of the BMI is of concern [3]. Many countries now have programs in place for educating and counselling prospective mothers to lose excessive weight before pregnancy and optimise the amount of weight gained during pregnancy. Dietary interventions combined with physical activity can reduce weight gain and lessen the need for caesareans [4]. During pregnancy, 50% of women exceed the recommended guidelines and the quality of the guidelines around ways to manage weight gain are poor [5]. Clearly, there is evidence of a gap to be addressed.

After giving birth, breastfeeding will be best for the baby’s development and to assist the mother to lose additional weight gain. There is also evidence suggesting that breastfed babies have a lower risk of becoming overweight at age two years and protection may indeed be offered into adulthood [6]. The rate of breastfeeding in the US at six months is about 56% [7], but more research, programs, and policies for improving these rates would be beneficial. Clearly, young children need sufficient nutrients for their growth and development, but their energy intake after weaning should not be excessive and weight gain should follow the percentiles of a healthy weight.

Early childhood centres have been a setting for programs and policy development to ensure healthy diets and the prevention of overweight and obesity. There have been numerous programs to encourage healthy eating, with or without the addition of physical activity, but the effectiveness of these interventions in improving children’s diets and preventing excessive weight gain remains questionable [8]. Fruit consumption may increase, but there is, overall, no impact on less healthy food consumption, nor on sugar-sweetened beverages [8]. Research into what other strategies may be needed to improve overall nutrition and prevent weight gain are suggested. Reducing sedentary screen time appears to be an important strategy for reducing early childhood obesity [9].

As children grow older, the school setting is an acceptable setting for lifestyle programs to prevent weight gain. Meta-analyses of interventions conducted since 2015 have demonstrated a small but positive effect on BMI [10]. Other settings such as after-school care or even home-based interventions have not demonstrated the same positive effects [10]. Further studies are indicated to strengthen the evidence base and design and test if effective interventions can be developed for families and communities. Research has shown the many factors around family mealtimes that may have positive influences. A greater family mealtime frequency may protect against obesity [11]. 

Adolescence is a time of rebellion, growing independence, and peer pressure. It is unsurprising that adolescents have poor-quality diets with a high consumption of sugar-sweetened beverages and ultra processed foods [12,13]. While many interventions have been conducted, especially in the school setting, further studies are indicated to strengthen the evidence base [14]. Adolescents are often seen as the most difficult age group to target with interventions, so innovation is needed. When young adulthood is attained, some of the food habits of adolescence remain, such as a high consumption of food prepared outside home and sugar-sweetened beverages [15]. This period can be a time of weight gain, with many becoming overweight in their twenties. Interventions aimed at improving healthy eating and physical activity have shown modest effects on the prevention of weight gain and weight loss [16]. Social support is often an important part of these interventions [16].

By mid-adulthood, most of the population in high-income countries is classified in the overweight and obese categories [17]. There have been a plethora of interventions aimed at the control of overweight and obesity and the prevention of further weight gain. Dietary patterns low in saturated fat and salt and rich in fruit, vegetables, wholegrains, legumes, and seafoods result in a lower BMI and waist circumference [18]. Weight loss interventions are successful, but maintaining weight after the initial loss proves difficult for most people. Interventions for the maintenance of weight loss should be an active area of research. We know that those successful at maintaining weight loss long-term have a higher physical activity and additionally eat more vegetables and less sugary and fatty foods. Having healthy foods available at home and eating breakfast are other characteristics associated with weight loss maintenance [19].

The prevention of weight gain must be based in a socioecological model [20]. The above personal, peer, community, and organisational interventions have been discussed, but it is at the policy level that interventions will impact the most people. Consistent with the NOURISHING framework, policy actions that address the food environment are needed as part of the efforts towards preventing harmful weight gain [21]. Policies around the regulation of outdoor food advertising and other marketing, especially to children, are needed. More research to assess the health impacts and effects on food consumption are indicated [22]. It is has been reported that, for every 10% increase in outdoor advertisements, 5% higher odds of obesity occur [23]. Also indicated is the regulation of food and menu labels. Menu labelling has demonstrated small to moderate effects on energy intake [24]. 

Economic tools are other instruments for creating a food environment conducive to healthy eating and the prevention of weight gain. The taxation of sugar-sweetened beverages has shown higher prices and the lowering of sales, but evidence on actual consumption is less clear [25]. The taxation of food according to its fat content has failed to show a positive effect on consumption [26]. It is uncertain whether taxing sugar-added foods would decrease intake or have any impact on the prevention of weight gain and obesity [27]. Further clarification of the role of taxation in food environments, consumption, and the prevention of weight gain appears necessary. 

Making healthy foods less expensive and price incentives are other means of improving the food environment and encouraging healthy eating. Interventions targeting price have been conducted in workplaces, tertiary institutions, and food outlets, with some success [28,29,30]. Other areas of intervention are food placement and nudging towards healthier choices [31].

While progress has been made in selected populations towards the prevention of weight gain, there is a need for further studies, and especially those for people who are disadvantaged, whether by education, financial opportunity, or belonging to a minority group. The prevention of harmful weight gain to halt obesity and its subsequent chronic diseases, such as diabetes, cardiovascular diseases, and some cancers, should remain high on health research agendas.
